# Preoperative ultrasound to identify distribution of the lateral femoral cutaneous nerve in total hip arthroplasty using the direct anterior approach

**DOI:** 10.1051/sicotj/2018037

**Published:** 2018-09-17

**Authors:** Yu Ozaki, Tomonori Baba, Yasuhiro Homma, Hiroki Tanabe, Hironori Ochi, Sammy Bannno, Taiji Watari, Kazuo Kaneko

**Affiliations:** Department of Orthopaedic Surgery, Juntendo University, 2-1-1 Hongo, Bunkyo-ku, Tokyo Japan

**Keywords:** Total hip arthroplasty, Direct anterior approach, Lateral cutaneous femoral nerve, Ultrasound, Quality of life

## Abstract

*Introduction*: Recently, the branching pattern of the lateral femoral cutaneous nerve (LFCN) named Fan type has been reported that LFCN injury cannot be avoided in surgical dissections that use the direct anterior approach to the hip joint in the cadaveric study. We hypothesized that the Fan type can be identified by ultrasound The aim of this study was to investigate whether LFCN injury occurs in DAA-THA in cases identified as the Fan type based on preoperative ultrasound of the proximal femur.

*Methods*: Ultrasonography of the proximal femur on the surgical side was performed before surgery and the LFCN distribution was judged as the Fan type or Non-Fan type. A self-reported questionnaire was sent to the patients at two months after surgery, and the presence or absence of LFCN injury was prospectively surveyed.

*Results*: After application of exclusion criteria, 45 hips were included. LFCN injury was observed after surgery in 9 of the 10 patients judged as the Fan type based on the ultrasound of the proximal femur (positive predictive value: 90%), and no LFCN disorder was actually observed in 25 of the 26 patients judged as Non-Fan type (specificity: 96.2%).

*Conclusions*: To prevent injury of the LFCN in patients judged as the Fan type on the ultrasound test before surgery, the risk of direct injury of the LFCN may be reduced through the approach in which an incision is made in the fascia which is opposite to the radial spreading, i.e., between the sartorius and tensor fasciae latae muscles or slightly medial from it.

## Introduction

Of approaches for total hip arthroplasty (THA), the direct anterior approach (DAA) is the only soft tissue-preserving approach using the inter-muscular and inter-nervous plane [1,2]. Functional discovery after surgery is fast compared with those after surgeries through the other approaches, and a low dislocation rate has also been reported [3–5]. Therefore, primary THA through the DAA has regained popularity in recent years.

However, DAA-specific complications have also been pointed out [6–8], and one of these is lateral femoral cutaneous nerve (LFCN) injury. The LFCN is a pure sensory nerve supplying the cutaneous area of the anterolateral thigh. Injury to the LFCN can result in hypesthesia or, in some patients, pain or dysesthesia in the anterolateral aspect of the thigh, and reduction of QOL due to LFCN injury after surgery despite the hip joint function being recovered by THA has been reported [9]. Risk factors of LFCN injury induced by DAA-THA include preoperative small femur offset [10]. It is considered that LFCN injury is caused not only by direct injury of the nerve but also by retraction with a retractor [10].

Regarding the distribution of the LFCN, many studies using ultra sound and cadavers have been reported, in which abundant distribution patterns were observed, showing individual variation [11–19]. In studies using ultra sound, it was stated that the LFCN or its branch in the proximal femur can be identified [17–19]. To understand the anatomy of the LFCN at the thigh level, Rudin et al. recently conducted a cadaveric study to examine the branching pattern of the LFCN and demonstrated three different branching patterns of the LFCN in the proximal aspect of the thigh [16]. They stated that of these patterns, the Fan-type nerve radially distributes in the transverse direction on the sartorius muscle over the tensor fasciae latae muscle and direct injury of the LFCN is unavoidable when a skin-fascia incision is made at a site lateral to the region between the sartorius and tensor fasciae latae muscles, which is currently employed in THA [16].

Thus, we hypothesized that the LFCN distribution in the proximal femur can be identified by ultrasound and LFCN injury develops in the Fan type, whereas small femur offset may be involved in LFCN injury in the types other than the Fan type. The aim of this study was to investigate: (1) whether LFCN injury occurs in DAA-THA in cases identified as the Fan type based on preoperative ultrasound of the proximal femur and (2) whether LFCN injury is likely to occur even in cases identified as Non-Fan type with a small femur offset before surgery.

## Materials and methods

**Figure 1:**
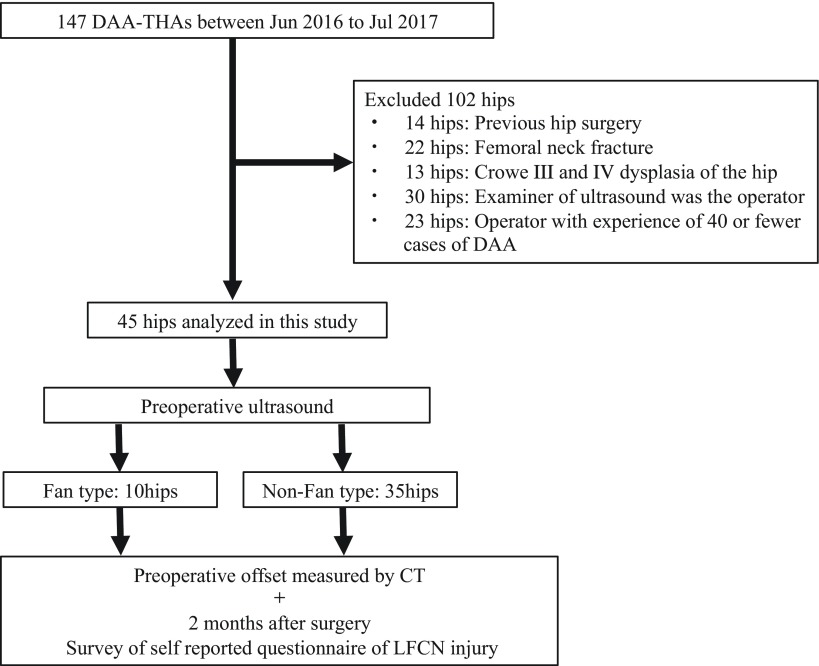
Flow chart of this study.

DAA-THA was performed in 147 hips between June 2016 and July 2017. Fourteen cases with previous of surgery of the hip joint on the affected side (including 8 cases of revision surgery), 22 cases of femoral neck fracture, 13 cases of Crowe III and IV developmental dysplasia of the hip, 30 cases in which the examiner of ultrasoud was the operator, and 23 cases operated by a hip joint surgeon with experience of 40 or fewer cases of DAA were excluded [8,20]. The remaining 45 hips which gave informed consent to the study before surgery as well as replied to a mail survey after the ultrasound test of the affected-side proximal femur were included in the study (Figure 1).

Ultrasonography of the proximal femur on the surgical side was performed before surgery and the LFCN distribution was identified as the Fan type or Non-Fan type based on the classification reported by Rudin et al. [16]. The operators performed surgery without information on the type of LFCN distribution in all cases. A self-reported questionnaire on postoperative LFCN injury was sent by postal mail to the patients after discharge, and the presence or absence of LFCN injury was prospectively surveyed [9,21]. LFCN injury was identified by symptoms of numbness, dull sensation, tingling or jolt-like sensation, pain, and/or a strange feeling over the lateral aspect of the thigh, excluding the surgical scar. Since spontaneous recovery from LFCN disorder 3–6 months after surgery has been reported, the questionnaire was sent 2 months after surgery [22,23]. In addition, cases identified as Non-Fan type based on the ultrasound were divided into groups with and without LFCN injury and the total offset, acetabular offset, and femoral offset were measured in CT images acquired for planning of surgery and compared [10,24] (Figure 1). Also in cases with LFCN injury, their offsets was compared between Fan type group and Non-Fan type group [10,24].

### Ultrasound

**Figure 2:**
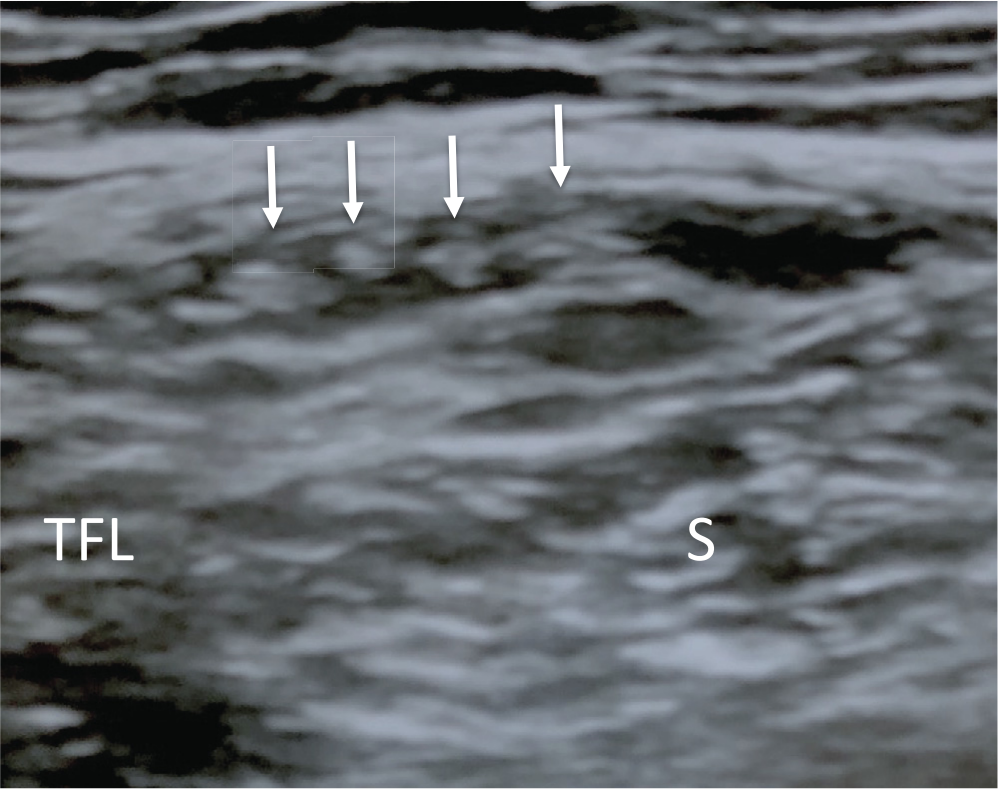
Transverse ultrasound image of the Fan type within the intermuscular space between the sartoirus and the tensor fascia latae. S: Sartorius, TFL: tensor fascia latae muscle, Arrows: several nerve branches.

**Figure 3:**
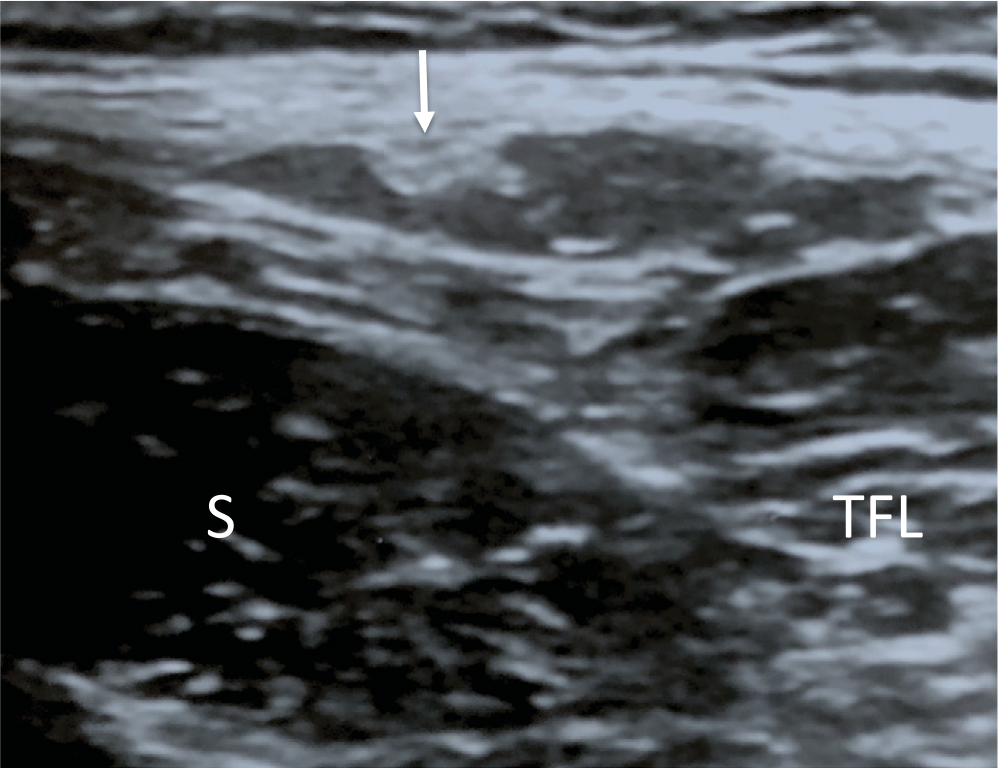
Transverse ultrasound image of the Non-Fan type within the intermuscular space between the sartoirus and the tensor fascia latae. S: Sartorius, TFL: tensor fascia latae muscle, Arrow: nerve branch.

All patients were positioned supine and were scanned on affected side using the LOGIQ e Expert (GE healthcare, Japan) with a 12 MHz linear array transducer. Ultrasonography was performed following the method reported by Zhu et al. [17]. The ultrasound transducer was placed in the transverse position and was first placed 1–2 cm distal to the lateral IL. Initially, the tensor fascia latae muscle and the sartorius were imaged. The LFCN was identified in the intermuscular space between the tensor fascia latae and the Sartorius [17]. The LFCN was identified and the probe was moved from the proximal to distal hip joint along the region between the sartorius and tensor fasciae latae muscle to confirm the LFCN distribution. An oval or spindle-shaped high-echoic region (epi- and perineurium) with an inner low-echoic region (nerve bundle) present between the sartorius and tensor fasciae latae muscle was identified as the LFCN or its branch. Following the definition made by Rudin et al. based on a cadaver study [16], several branches with a similar size present between the muscles were defined as the Fan type (Figure 2). The main root or major branch with surrounding thin branches was defined as the Non-Fan type (Figure 3).

### Preoperative offset measurements

**Figure 4:**
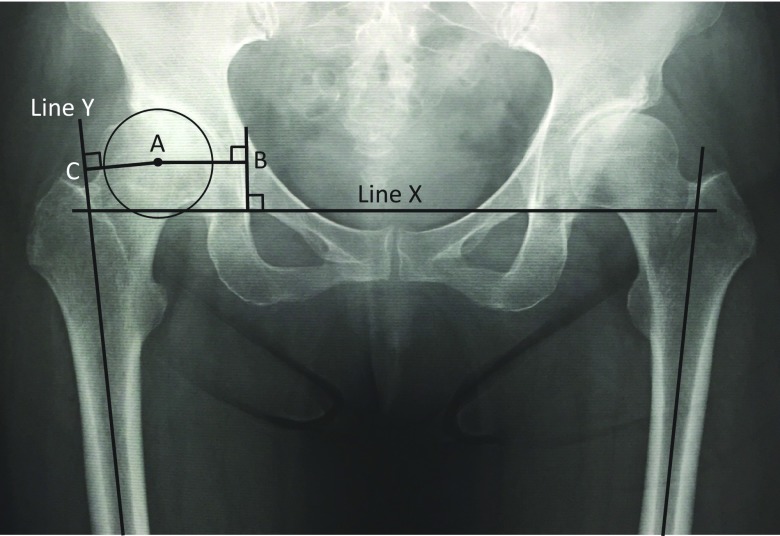
An anteroposterior radiograph of the hips was obtained in a supine position immediately preoperatively. X: trans-teardrop line; Y: central axis of the femur; Z: central axis of the femoral neck; point A: center of the femoral head; AB: acetabular offset; AC: femoral offset. The total offset is the sum of lines AB and AC [10].

As reported previously, a preoperative CT was used in place of the X-ray to obtain more precise offset measurements according to Pasquier et al. [24]. Therefore the coordinates of the center of the femoral head were obtained by superimposing circles of various sizes on the head, and the diameter of the circle that corresponded most closely to the edges of the femoral head was taken to be the diameter of the femoral head [24]. Femoral offset was defined by a perpendicular line drawn between the anatomical femoral axis and the center of the femoral head. Acetabular offset was defined by a perpendicular line drawn between the center of the femoral head and a line through the teardrop, perpendicular to the trans-teardrop line (Figure 4). The coordinates of the center of the femoral head were defined for axial and coronal planes of reference in the preoperative CT. Acetabular and femoral offsets were measured using the coronal plane.

### Surgical technique

All surgeries were performed by 3 hip joint surgeons with experience of 40 or more cases of THA through DAA [8,20]. DAA was performed with the patient in the supine position on a standard surgical table. The skin incision began 2 cm lateral and distal to the anterior superior iliac spine and proceeded distally for up to 10 cm along a line angling toward the head of the fibula. Briefly, the fascia of the TFL muscle was incised about 2 cm lateral to the skin incision to prevent LFCN injury, and the intermuscular space between the TFL and sartorius muscles was bluntly entered. The anterior articular capsule was exposed, incised, and resected as much as possible to expose the femoral head. For stem insertion, the surgical table was extended so that the hip joint could be extended to 15°. The superior and posterior portions of the articular capsule were partially incised so that the greater trochanter could be elevated with a retractor. Finally, the size and stability of the implant were confirmed intraoperatively under fluoroscopy.

### Statistical analysis

We used the Mann–Whitney U test to compare differences in each preoperative offset between patients reporting LFCN injury, versus those with no symptoms. Differences with a *p*-value < 0.05 were considered statistically significant. All statistical analyses were performed using IBM SPSS Statistics for Mac IBM Corp., Armonk, NY, USA).

## Results

We examined the proximal femur of the 45 hips by the ultrasound test before surgical treatment with THA through DAA and surveyed the presence or absence of LFCN injury 2 months after surgery.

There were 12 males and 33 females, with a mean age at surgery of 65.3 years (range 42–90 years). Osteoarthritis was diagnosed in 35 hips (%), osteonecrosis in eight hips (%), and Rheumatoid arthritis in two hips (%). There were 18 procedures involving the right hip (40%). The mean body mass index was 24.2 kg/m^2^ (range 18–35 kg/m^2^). LFCN injury occurred in 19 (42.2%) of the 45 hips (Table 1). Ten hips were identified as the Fan type based on the preoperative ultrasound, and 9 (90%) of these developed LFCN injury after surgery (Table 1). Thirty-five hips were identified as Non-Fan type and 10 (28.6%) of these developed LFCN injury (Table 1). The sensitivity was 47.4%, the specificity was 96.2%, the positive predictive value was 90%, and the negative predictive value was 71.4%. In the 35 hips identified as Non-Fan type, the femur offset was significantly smaller in the group with than without LFCN injury (group with vs. without LFCN injury: 27.7 ± 5.6 vs. 32.2 ± 5.1 mm, *p* = 0.03) (Table 2). On the other hand, no significant difference was noted in the acetabular offset (40.7 ± 6.9 vs. 38.0 ± 5.2 mm, respectively, *p* = 0.21) and total offset (67.5 ± 6.9 vs. 70.2 ± 8.0 mm, respectively, *p* = 0.35) between the 2 groups (Table 2). In the 19 hips with LFCN injury, the femur offset was significantly smaller in the Non-Fan type group than the Fan type group (Fan type vs. Non-Fan type: 32.2 ± 2.7 vs. 27.7 ± 5.6 mm, *p* = 0.047) (Table 3). no significant difference was noted in the acetabular offset (38.1 ± 5.1 vs. 39.7 ± 6.6 mm, respectively, *p* = 0.54) and total offset (70.3 ± 5.5 vs. 67.5 ± 7.0 mm, respectively, *p* = 0.35) between the 2 groups (Table 3).

**Table 1:** Result of preoperative ultrasound and survey of self reported questionnaire of LFCN injury.

	LFCN injury (+)	LFCN injury (−)	Total
Fan type	9	1	10
Non-Fan type	10	25	35
Total	19	26	45

LFCN: lateral femoral cutaneous nerve.

**Table 2:** Result of preoperative offsets of patients with and without lateral femoral cutaneous injury in Non-Fan type.

Variables	LFCN injury (+)	LFCN injury (−)	*p* value
	(*n* = 10)	(*n* = 25)	
Femur offset (mm)	27.7 ± 5.6	32.2 ± 5.1	0.03^*^
Acetabular offset (mm)	40.7 ± 6.9	38.0 ± 5.2	0.21
Total offset (mm)	67.5 ± 6.9	70.2 ± 8.0	0.35

LFCN: lateral femoral cutaneous nerve.

**Table 3:** Result of preoperative offsets of Fan type and Non-Fan type in patients with lateral femoral cutaneous nerve injury.

Variables	Fan type	Non-Fan type	*p*-value
	(*n* = 9)	(*n* = 10)	
Femur offset (mm)	32.2 ± 2.7	27.7 ± 5.6	0.047^*^
Acetabular offset (mm)	38.1 ± 5.1	39.7 ± 6.6	0.54
Total offset (mm)	70.3 ± 5.5	67.5 ± 7.0	0.35

LFCN: lateral femoral cutaneous nerve.

## Discussion

Since the DAA for THA is a soft tissue-preserving approach using the inter-muscular and inter-nervous plane, functional discovery after surgery is fast compared to those after surgeries through the other approaches, and the dislocation rate is low, being superior in many points [3–5]. However, the only concern, LFCN injury, is specific to the DAA, and this complication leads to reduction of quality of life [9]. In the proximal femur, the LFCN either crosses the intermuscular space between the tensor fascia latae (TFL) and sartorius muscles or lies within it. Because the surgical interval for the DAA is the intermuscular space between the TFL and sartorius muscles, the LFCN is always in the vicinity of the surgical field and is at risk of injury [5,12,22].

We consider that acquisition of ability to identify the LFCN distribution before surgery is the first step to prevent anterior approach-induced LFCN injury and this is also useful to take preventive measures in the future. Thus, focusing on non-invasiveness of ultrasonography, we tried to identify the LFCN distribution in patients before THA. LFCN injury was observed after surgery in 9 of the 10 patients identified as the Fan type based on the ultrasound of the proximal femur (positive predictive value: 90%), and no LFCN injury was actually observed in 25 of the 26 patients judged as Non-Fan type (specificity: 96.2%). These findings suggested that when the LFCN distribution on the ultrasound test is identified as the Fan type before surgery, LFCN injury may develop after surgery at a high probability. Rudin et al. reported that there were 3 main patterns of the LFCN distribution in the proximal femur in a cadaveric study [16]. In the DAA currently employed in many cases, to avoid LFCN injury, the skin is incised at a site slightly lateral from the region between the sartorius and tensor fasciae latae muscle, i.e., right above the muscle belly of the tensor fasciae latae muscle, and dissection is advanced toward the region between the 2 muscles [25]. However, Rudin et al. stated that the nerve branches radially while distributing in the transverse direction between the muscles in the Fan type and direct injury of the LFCN is unavoidable using this approach in this type [16], strongly supporting our findings. To prevent injury of the LFCN in patients identified as the Fan type on the ultrasound test before surgery, further devising of the approach may be necessary. Normally, to prevent injury of the LFCN, it is recommended to incise the fascia at a site slightly lateral from the region between the sartorius and tensor fasciae latae muscle and advance dissection into the hip joint [25], but the risk of direct injury of the LFCN may be reduced through the approach in which an incision is made in the fascia which is opposite to the radial spreading, i.e., between the sartorius and tensor fasciae latae muscles or slightly medial from it.

Symptoms of LFCN injury were observed in about 29% of the Non-Fan-type cases that should be able to avoid LFCN injury. The femoral offset was significantly smaller in these cases with LFCN injury. In addition, their offset was also significantly smaller than the Fan type cases with LFCN injury.

They are indicated that not only the problem with the LFCN distribution, such as the Fan type, but the femoral offset has an influence on the developmental mechanism of LFCN injury, as previously reported [10]. Several mechanisms have been described for LFCN injury, including nerve stretching, compression, laceration, and involvement in scar tissue formation [5,22]. Of these, nerve stretching may be most frequently involved in LFCN injury in the Non-Fan type because stretching is strongly influenced by not only the type of LFCN distribution but also the femoral offset [10]. The DAA requires that the sartorius and tensor fascia latae muscles be separated. Ropars et al. mentioned that use of retractors into the intermuscular space between Sartorius and tensor fascia latae can result in stretching of the branch along this intermuscular space [12]. In the current situation, a retractor with the same size is used with a maximum force in this working space in all patients. Therefore, we consider that a large tension relative to the soft tissue including the LFCN is loaded in patients with a small femoral offset, increasing the probability of causing LFCN injury [10].

There are several limitations of this study. Firstly, the sample size was small. Secondly, only one examiner performed ultrasonography, so that validity, such as the concordance rate among examiners, could not be evaluated. Thirdly, the presence of LFCN injury was identified based on only subjective evaluation in the questionnaire. No objective evaluation or survey of neurologic manifestation derived from lumbar vertebral disease as differential diagnosis was performed. However, no previous study on LFCN injury performed the echo test before surgery or verified the clinical outcomes after surgery. Thus, we believe that the present study is sufficiently valuable as a preliminary study aiming at reduction of these complications.

## Conflict of interest

The authors declare that they have no conflict of interest.
